# Improved Face Detection Method via Learning Small Faces on Hard Images Based on a Deep Learning Approach

**DOI:** 10.3390/s23010502

**Published:** 2023-01-02

**Authors:** Dilnoza Mamieva, Akmalbek Bobomirzaevich Abdusalomov, Mukhriddin Mukhiddinov, Taeg Keun Whangbo

**Affiliations:** 1 Department of Computer Engineering, Gachon University, Sujeong-gu, Seongnam-si 461-701, Gyeonggi-do, Republic of Korea; 2 Department of Artificial Intelligence, Tashkent State University of Economics, Tashkent 100066, Uzbekistan

**Keywords:** face detection, retina net, region offering network, deep learning

## Abstract

Most facial recognition and face analysis systems start with facial detection. Early techniques, such as Haar cascades and histograms of directed gradients, mainly rely on features that had been manually developed from particular images. However, these techniques are unable to correctly synthesize images taken in untamed situations. However, deep learning’s quick development in computer vision has also sped up the development of a number of deep learning-based face detection frameworks, many of which have significantly improved accuracy in recent years. When detecting faces in face detection software, the difficulty of detecting small, scale, position, occlusion, blurring, and partially occluded faces in uncontrolled conditions is one of the problems of face identification that has been explored for many years but has not yet been entirely resolved. In this paper, we propose Retina net baseline, a single-stage face detector, to handle the challenging face detection problem. We made network improvements that boosted detection speed and accuracy. In Experiments, we used two popular datasets, such as WIDER FACE and FDDB. Specifically, on the WIDER FACE benchmark, our proposed method achieves AP of 41.0 at speed of 11.8 FPS with a single-scale inference strategy and AP of 44.2 with multi-scale inference strategy, which are results among one-stage detectors. Then, we trained our model during the implementation using the PyTorch framework, which provided an accuracy of 95.6% for the faces, which are successfully detected. Visible experimental results show that our proposed model outperforms seamless detection and recognition results achieved using performance evaluation matrices.

## 1. Introduction

One of the most well-known, essential, and practical issues in computer vision systems is face detection, the objective of which is to extract information from facial images. Face detection is an essential first step in face verification [1], face identification and clustering [2], facial landmarks [3], facial hallmark classification [4], face alignment [5], and face tracking [6]. The goal of Viola–Jones’ study was to develop an object detection framework [7] in 2001. Over the past ten years, numerous face-identification techniques have been developed. Despite notable advancements made in the field over the course of the last couple’s protracted decades, accurate and effective face identification in the wild has continued to be a challenge. The face identification depends on variables in position, face occlusion, scale, lighting, image infringement, facial expressions, and other elements. Face detection differs from typical object detection; it has smaller component ratio variations, but considerably larger scale alterations that range from several pixels to thousands of pixels.

The conventional strategy, which extracts constructed capabilities from the image and uses several classifiers to almost certainly pinpoint face regions, was the foundation for early face detection efforts. In addition, the Haar cascade classifier [7] and histogram of oriented gradients (HOG) followed by the support vector machine (SVM) [8] are two other important landmark classical studies for face detection. These studies represent the most recent state-of-the-art achievements. However, the accuracy of face detection in difficult images with unresolved variations continues to be limited in the WIDER FACE facial detection dataset [9].

Deep learning, specifically deep convolutional neural networks (CNN), has proven highly successful in recent years in a variety of computer vision applications [10], including image classification, object detection, semantic segmentation, and deep learning algorithms, which skip the hand-crafted design pipeline and have control over several well-known benchmark evaluations, such as the ImageNet Large Scale Visual Recognition Challenge (ILSVRC) [11]. This is in contrast to traditional computer-vision systems.

A surge in research interest to investigate deep learning for addressing face detection challenges has been developing, owing to the growing prominence of deep learning in computer vision. Object detection has recently made excellent progress [12,13,14], taking inspiration from common object detection techniques [15,16,17] that have incorporated all the current deep learning advancements. Therefore, face detectors can achieve much better detection results than conventional cascaded classifiers using Faster R-CNN [15], YOLO [16], or single shot detector (SSD) [18]. There are several comparable works, such as Face R-CNN [19] and Face R-FCN [20], which have been improved and adjusted using R-FCN and Faster R-CNN [21]. Additionally, certain other detectors, such as multitask cascaded convolutional networks (MTCNN) [22,23], had considerable success owing to the significant and state-of-the-art benefits over WIDER FACE [9] while achieving 16 frames per second on a 2.6 GHz CPU. The multiscale mechanism from SSD [17], feature enhancement from FPN [24], and focus loss from RetineNet [25] have all been modified from common object identification approaches to be used for face detection in accordance with the unique pattern of images of human faces. These methods led to the proposal of different outstanding face detectors, such as S3FD [26], Pyramid Box [27], SRN [28], DSFD [29], and Retina Face [30]. Additionally, the latest state-of-the-art techniques [12,14] emphasize single-stage [17] design, in which density samples are placed in areas and scales of feature pyramids, exhibiting promising performance and submitting data more quickly than two-stage approaches [12,31].

By introducing a branch for concurrently predicting an object mask based on the existing branch for bounding box detection and regression in Mask R-CNN [32], this demonstrates the value of extensive pixel-level annotations for enhancing detection. Unfortunately, dense face annotation is not possible for the hard face images of WIDER FACE. Recently, the WIDER face dataset [9] contains a large number of tiny faces, exposing the implementation difference between people and present face detectors. This problem becomes more difficult if the speed and memory productivity of the detectors are considered. The best-performing face detectors are commonly not fast and have large memory footprints, partly because of the large number of parameters and the way robustness to scale or corporation of context is addressed.

The remainder of this paper is organized as follows: Section 2 reviews existing studies on face detection. In Section 3, the proposed face detection method is described in detail. Section 4 presents experimental findings, and Section 5 concludes the paper and discusses the future directions of the proposed method.

## 2. Related Work

In this section, we discuss a number of methods that have been utilized for face detection and recognition over the years, from early techniques to modern techniques. Various techniques for face detection and identification have emerged during the last few years. Four primary object detection algorithms can be used to combine all face detection techniques. [33]

Computer vision systems have been extensively researched in early face-detection studies [34]. Despite many extensive studies in recent years, based on the technique proposed by Viola–Jones [7], there exists insufficient practical results on face detection. The VJ framework [7] was one of the first frameworks to achieve real-time face detection by applying rectangular haar-like features to a cascaded AdaBoost classifier. However, these methods are not end-to-end trained, and feature learning and classifier training are trained separately. We can obtain the right running speed, but not sufficient accuracy. Although a good running speed is obtained; it does not have a satisfactory accuracy. SVMs [35] can be trained for face detection, a good example of which is the Haar wavelet. When Haar wavelets are trained on positive and negative applied examples of feature extraction, it helps to distinguish the classes; however, they faced a problem—it could not pick up the faces of various poses because they were weak, which resulted in the poor performance of the classifier and the results were indeterminate. Haoxiang Li et al. [36] proposed a CNN cascade using different resolutions, where the background area was discarded in the fast, low-resolution phase, and some difficult decisions were carefully evaluated in the final, high-resolution phase. Using the CNN-based calibration after each detection stage in the cascade increased the localization and decreased the number of candidates for the later stages. The status of the detection window is normalized using the output of each calibration stage as an input to the following calibration stage. The CNN-based techniques present facial detection methods and inherit some achievements from the commonly existing techniques. These fall into two categories of approaches: two-stage (faster R-CNN [31,37]) and one-stage (SSD [18,26]; and Retina Net [14,17]).

The two-step approach uses a highly accurate “offering and improving” mechanism for localization. In contrast, the one-step method carefully samples facial positions and scales to derive true and false samples without training principles. The sampling [38] and reweighting [13] techniques are widely used to reduce this imbalance. Compared to the two-step method, the one-step method is very productive and has a very high recall, but is at the risk of higher false-positive rates and less accurate localization.

Recently, the MTCNN [21] performed face detection using a sliding window method and relied on an image pyramid. HR [39] is a multilevel variant of the MTCNN that also requires an image pyramid. The image pyramid has several drawbacks: it is not fast but has a high speed and large memory rate. It considers HF when designing the discovery branch and provides an anchor-matching technique to improve hit costs. In [40], Zhu et al. focused on small-face detection by providing powerful anchor generation and matching techniques. We conclude that anchor-related techniques are important for face recognition. Subsequently, S3FD [15] and Pyramid Box [17] enhanced the backbone based on the low-level functional pyramid layer (LFPN) to improve various detections. SSH [23] builds three cognitive architectures that work together based on a contextual architecture for scale-invariant face detection.

DSFD [28] features enhanced modules, forward layer monitoring, and improved anchor matching procedures for quick initialization. S3FD, pyramid box, SSH, and DSFD use VGG16 as the backbone, which results in a large architecture size and unproductive computation. Facebox [41] works by drastically shrinking the size of the input face image to run face detectors in real-time. After four layers consisting of two layers of convolution and two layers of pooling, a large step size of 32 was reached. Faceboxes are fast but have limited accuracy due to their inability to detect small facial images. For the face detection, we use RetineNet architecture as mentioned earlier. All identified faces are recognized using region offering network (RON) and high feature generation pyramid (HFGP), low feature generation pyramid (LFGP), and we trained our model during the implementation using the PyTorch framework which provided an accuracy of 95.6% for the faces which are successfully detected.

## 3. Proposed Face Detection Method

**Figure 1:**
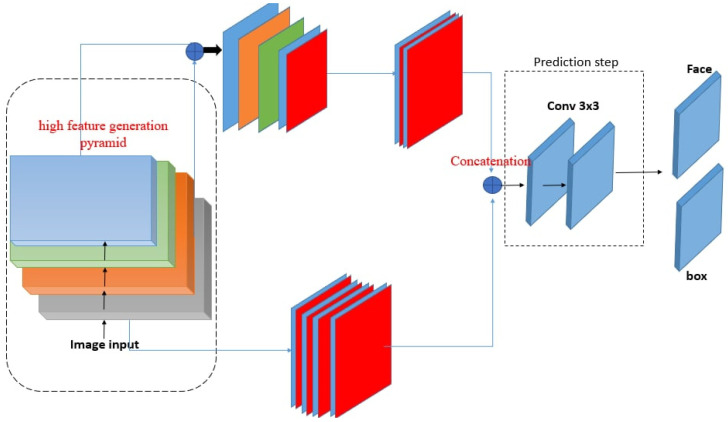
Architecture of proposed method for face detection.

The proposed method uses RetinaNet’s deep learning framework, which is an advanced deep learning design for common object detection. It is crucial to have two parts: (1) a region offering network (RON) to compile a list of area suggestions that almost certainly include faces, and (2) a prediction branch for identifying faces in an area of the image and fine-tuning the boundaries of these areas. This model can perform face image detection at a competitive speed because of the elements contributing to the general parameters for the convolution layers employed in feature extraction. In this work, we suggest improving the recall and accuracy of facial image detection using the Retina Net architecture and train our face detection model with the aid of following the proposed systems show in Figure 1. First, we trained the model of RetinaNet [14] using the Wider Face dataset [9]. We also tested the pre-trained model using the same dataset to ensure that it produces hard negatives. As the second step of our training technique, these hard negative examples are sent into the network. By training on these hard negative samples, the resulting model is capable of producing fewer false positives. We used the FDDB dataset to further fine-tune the process in our method [42]. However, as this dataset only contains 5171 faces in 2845 images, merely fine-tuning it may not be the smart choice. In our method, we first pre-trained our model on the wider face dataset, a considerably larger face dataset with much more challenging cases, before fine-tuning it on FDDB. Additionally, we used the multi-scale training processes during the final fine-tuning stage. We adopted a similar end-to-end training methodology to RetinaNet because of its effectiveness and simplicity. As a final optional step, we transformed the obtained detection bounding boxes into rectangular regions of human faces. In the following, we discuss five key steps of our solution in detail.

### 3.1. Feature Extraction—Region Offering Network

Our network consisted of three parts. First, to create the foundation feature, the high feature generation pyramid (HFGP) combines shallow and deep features. For instance, conv4 3 and conv5 3 of ResNet ensure multilevel semantic pieces of information for feature maps. Second, a low-feature-generation pyramid (LFGP) and convolution layers are stacked alternately. In particular, LFGP generates low-level feature maps with a scale different from that of the HFGP. The convolution layers combine the main features and the large output feature map of the preceding pyramid-based layers. In addition, the added feature maps were supplied to the next convolution layer. These layers of convolution study the properties from the layers of the pyramid and take them as the basic properties of $F_{base}$. The output multi-scale features are calculated as follows:

(1) $$\left[f_{1}^{l},f_{2}^{l},\ldots ,f_{i}^{l}\right]=\{\begin{array}{c} T_{l}\left(F_{base}\right),\\ T_{l}{}_{\left(P\left(F_{base,\;f_{i}^{l-1}}\right)\right),} \end{array}\;\begin{array}{cc} l=1\; & \\ l=2\ldots L^{\prime } & \end{array}$$

where in Fbase $F_{base}$ denotes the feature, $f_{i}^{l}$ denotes the feature with the i-th scale within the l-th LFGP, $T_{l}$ denotes the l-th HFGP processing, and P denotes HFGP processing. Third, Prediction Step (PS) aggregates the multi-stage, multi-scale features by means of a scale-sensible feature concatenation operation, and a channel sensible attention mechanism.

### 3.2. High Feature Generation Pyramid (HFGP)

HFGP fuse feature from one level in our network, which is essential for creating the last multi-degree feature pyramid. They used the channels of the input features 1 × 1 convolution layers for compression and coupling operations can be used to combine these feature maps. In particular, because HFGP takes feature maps with one-of-a-kind scales in the backbone as input, it accepts the one up sample operation to rescale the deep functions to the same scale as the coupling operation. Taking HFGP from very deep backbone features results in stronger detection; therefore, high decision prototypes bring about better functional extraction and good work on small objects.

### 3.3. Low Feature Generation Pyramid (LFGP)

LFGP is different from HFGP and RetinaNet. The pyramid network consists of a chain of 2-stride 3 × 3 convolution layers. Then, the convolution layers use the outputs of those layers as their information set for the feature maps. The lower convolution layer in the HFGP backbone selects the final layer at every level. To enhance the learning ability and maintain feature smoothness, we also added 1 × 1 convolution layers after the up sample and detailed the clever sum working within the top convolution layer network.

The outputs from every convolution layer in the HFGP and LFGP were combined to provide multi-scale characteristics of the present level. Overall, the outputs of the stacked LFGP create multi-degree, multi-scale features, with the front LFGP imparting shallow-level, middle-level, then returning LFGP to ensure deep-level features.

### 3.4. Prediction Step

Prediction Step (PS) aims to combine the multi-degree and multi-scale characteristics brought about by LFGP and HFGP into a convolution layer. Connecting functions of the same scale collectively over the channel dimension are the initial step in the *PS*. The aggregated function pyramid can be expressed as F = $\left[F_{1,}\;F_{2,}\ldots F_{i,}\right]$ where $F_{i}=Concat\;\left(x_{i}^{1},\;x_{i}^{2},\ldots ,x_{i}^{L}\right)\in R^{Wi\times Hi\times C}$ refers to the features of the i**-** th large-scale. Every scale inside the aggregated pyramid includes capabilities from multilevel depth. Moreover, easy coupling operations are not sufficiently adaptive for the prediction head devoted to every feature, and we have one 3 × 3 Conv contribution via all three networks, after which every network takes its own 3 × 3 Conv in parallel. Our prediction head model is extremely lightweight and quick compared with RetinaNet. Additionally, we trained class prediction using OHEM [43] with a 3:1 neg pos ratio, softmax cross-entropy, c positive labels, and one background label. As a result, unlike RetinaNet, we did not use focus loss, which we have demonstrated to be insignificant in our case.

### 3.5. Concatenation

To promote the recognition of features on channels where they are most advantageous, we proposed a channel-specific attention module. Following the PS block, we used channel-wise statistics $z\in R^{C}$ in the compression step using global middle pooling. The following agitation stage learns the attention mechanism using two convolution-related layers to fully capture channel-wise dependencies:

(2) $$s=P_{ex}\left(z,\;W\right)=\sigma \left(W_{2}\sigma \left(W_{1}z\right)\right)$$

where σ is the ReLU function, δ refers to the sigmoid function, $W_{1\;}\in \;R^{C\times \frac{C}{r}\;}$,

$W_{2}\in \;R^{C\times \frac{C}{r}\;},\;r$ is the reduction ratio (r = 16 in our experiments). The final output is obtained by reweighting input F with activation s:

(3) $${\tilde{F}}_{i}^{c}=P_{scale}\left(F_{i}^{c},s_{c}\right)=s_{c}\cdot F_{i}^{c}$$

where $[\tilde{F}._{i}=\tilde{F}._{i}^{1}$, $\tilde{F}._{i}^{2},\ldots \tilde{F}._{i}^{C}],$ each of the features is enhanced or weakened by the rescaling operation.

## 4. Implementation and Results

In this section, we present experiments on challenging dataset from WIDER FACE bounding box detection challenge. We followed the WIDER FACE protocol of having total faces in images with different detection difficulties, such as occlusions, hard poses, out-of-focus faces, and low resolution. For a comparison based on state-of-the-art techniques, we reported open-access face datasets on the test-dev split, which is generally available, labelled, and does not demand the application of the assessment server. Then, we describe the results of the ablation learning assessed on the minimal split for comfort.

### 4.1. Implementation Details

We trained our model during the implementation using the PyTorch framework [44]. Table 1 of ResNet 50 was selected as the backbone of our CNN network, which was pre-trained on Image Net. The WIDER FACE training and validation datasets were used as the training datasets in the first stage. We provide a hard value for each ground-truth annotation in accordance with the level shown in Table 1. Specifically, zero issues were used as the initialization basis for all faces. The location satisfied the positive direction stated in Table 1, along with the face. Then, we proceeded to add an appropriate hard value. Additionally, we did not consider annotations whose difficulty values were greater than 2.

Then, all images that were based on more than 1000 annotations were also thrown out, as in our previous studies [45,46,47,48,49]. On the abovementioned dataset, the pre-trained ResNet architecture was trained using 200 iterations with a learning rate of 0.0001. The images were first resized in this training procedure while maintaining the original party ratio. The longer aspects were capped at 1000, and the shorter aspects were rescaled to 600. Horizontal flipping was used for data augmentation. For the region offering network branch, 12 anchors were employed in the training process, covering a total size of 64 × 64, 128 × 128, 256 × 256, and 512 × 512, respectively, and three aspect ratios: 1:1, 1:2, and 2:1, respectively. Following the non-maximum suppression (NMS), 2000 region offers are retained. The second step is fed into the network using the aforementioned dataset.

The “hard negatives” are those output locations with confidence ratings more than 0.8 and IoU values with any ground-truth annotation less than 0.5. Additionally, using a fixed learning rate of 0.0001, the difficult negative mining technique was run for 150 iterations, after which it was ensured that those difficult negatives were selected together with the various sample images. To produce our final detection model, we fine-tuned the resulting model using the FDDB dataset. We performed a series of five-fold cross-validation experiments to examine the detection design of our model on FDDB. We randomly resized each face image before placing it into the network to achieve horizontal flipping.

We scale every face image such that one in every 480, 600, and 750 pixels, respectively, will be its shorter aspect. In addition, we ensured that the longer issue did not surpass 1250, similar to the coverage taken in the first step. We used a feature concatenation approach to add the features pooled from the conv3 3, conv4 3, and conv5 3 layers during the training process. The scale was utilized once the features added the potential to be improved upon or fixed. Additionally, for both the training and test stages, we applied a fixed scale of 4700 to the entire blob. We used our final model after 80 iterations of architecture within a fixed learning rate of 0.001.

Next, the test period was examined after resizing a query face image using the same method as in the first level [50,51]. The region offering network branch network in the region offers a generating stage that generates 100 region offers for every facial image. If the trust rating of the classification is greater than 0.8. A chosen region was considered as a face. The non-maximum suppression limit in our study was set to 0.3. In our experiments, we also output all-region recommendations with trust degrees greater than 0.001.

In addition, on a computer with two NVIDIA Titan X GPUs, CUDA 9.2, and cuDNN 7.1.4, we trained the network on an NVIDIA Tesla V100 to obtain results temporarily and quickly. The set batch size is 32. Thus, the training method is restricted to the 12 GB memory NVIDIA Titan Xp if the batch size on a single GPU is less than 5.

In our model based on the VGG-16 backbone, the complete training time rates are three and six days for input sizes of 320 × 320 and 512 × 512, respectively, and for the ResNet-101 backbone, 512 × 512 costs five days.

### 4.2. The Process Speediness 

We also evaluated the inference speed of our model using state-of-the-art techniques. It is quick to apply VGG-16 for extracting base features because it has removed FC layers and makes less of a backbone. Then, with the batch size set to 1, we computed the inference time for each image by adding the CNN and NMS run times for 1000 face images and dividing by 1000. We suggest a fast version with an input size of 320 × 320, and a standard version with an input size of 512 × 512 and reduced VGG16, which is reduced to the proposed method. Based on the PyTorch optimization, our model can quickly produce accurate results. This work benefits from Table 2 by demonstrating the superiority of one-stage detection, and the multilevel structure of this method yields a very clear and positive speed-accuracy curve when compared to other approaches. Additionally, we replicated and tested the speed using additional techniques on our device for comparison.

**Figure 2:**
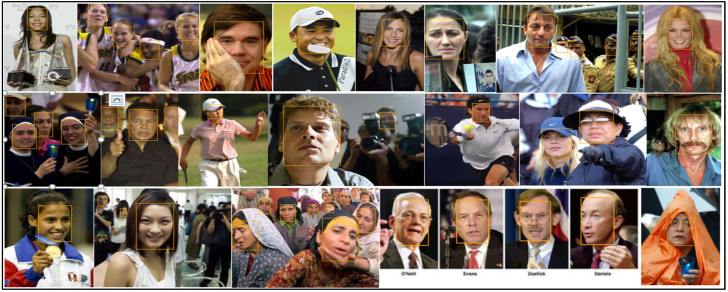
The detection results the FDDB dataset.

**Figure 3:**
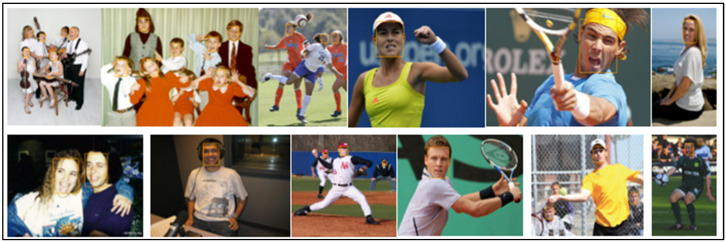
Expression image results (WIDER FACE dataset).

**Figure 4:**
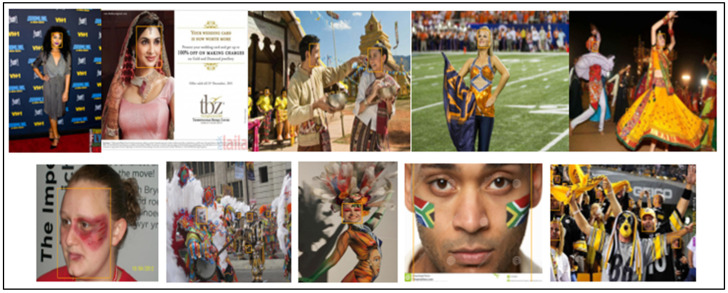
Makeup image results (WIDER FACE dataset).

**Figure 5:**
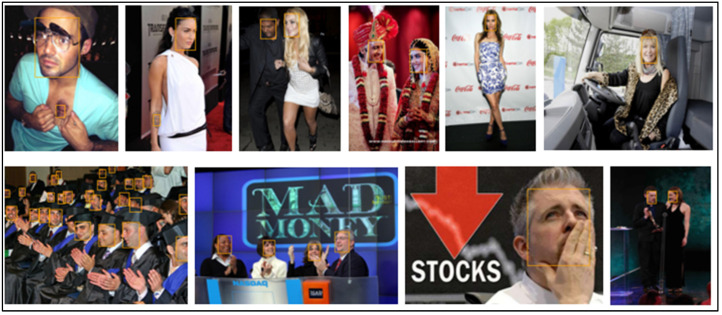
Occlusion image results (WIDER FACE dataset).

**Figure 6:**
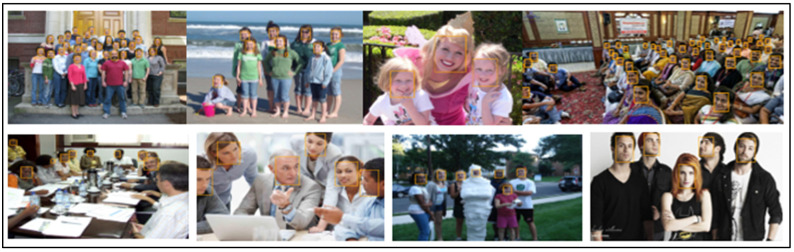
Pose images results (WIDER FACE dataset).

**Figure 7:**
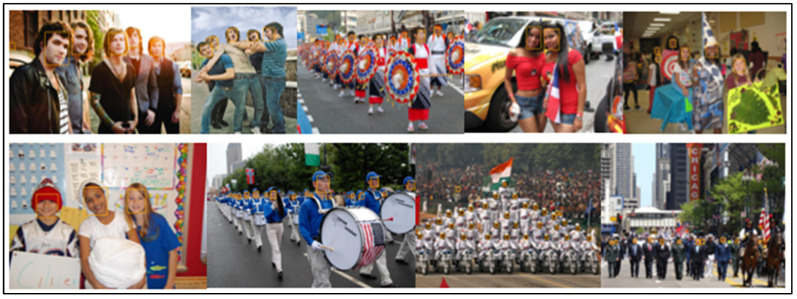
Scale images results (WIDER FACE dataset).

Table 3 lists a thorough comparison of the few published competitive strategies used for the WIDER FACE benchmark. To further demonstrate the efficiency of the proposed method for face detection based on deep learning methods, we randomly selected qualitative outcomes of face detection instances for various situations, as shown in Table 3. This shows how well our suggested model can identify and find dissimilar cases, such as faces that are hard occluded, in unusual positions, illumination, etc. A few false negatives are included in the list, which includes a few challenges, including small, blurry, and closely occluded faces. Table 3 lists the test results for the proposed model with ten distinct configuration versions. The batch size was set to 1, and only one NVIDIA Titan X PASCAL was used. The FDDB test-dev split was used for the testing. References provided additional statistical findings. Note that our proposed model, which uses a VGG backbone, has an AP of 38.9, outperforming competing object detectors that have extremely robust backbones and sizable inputs. For instance, the AP of the deformable R-FCN was 37.5, and the AP of R-CNN with FPN was 36.2. ResNet-101′s single-scale version has an AP of 38.8, which is comparable to modern two-stage detectors, such as mask R-CNN. Accumulation with ResNet-101 increased our results. Additionally, owing to PyTorch’s optimization, it can operate at 15.8 frames per second (FPS). RefineDet receives an AP of 41.8 and gains the advantages of both one-stage and two-stage detectors; whereas CornerNet suggests key point regression for detection and gains the advantages by doing so, earning an AP of 42.1. In contrast, our proposed method, which takes 44.2 AP and outperforms all one-stage detectors, is based on the regression algorithm of the original SSD and supports multi-scale multi-level features. We only evaluated the speed of the single-scale inference method owing to the range of tools or methods used; most approaches do not compare the speed of multiscale inference strategies. Based on the state-of-the-art, we also contrast one- and two-stage detectors, which can indicate that the development of the proposed model is not solely due to the increased depth of the model or the added parameters. Mask R-CNN with ResNeXt-101-32 × 8d-FPN has 205 M parameters, whereas CornerNet with Hourglass has 201 M parameters. In contrast, the proposed model VGG only contains 147 M parameters. Additionally, it was not dominant when comparing depths. Experimental results indicated that our improved face detection method accurately detected face regions. In addition, our method works effectively, even when there are multiple faces in the frame sequences, as shown in Figure 2, Figure 3, Figure 4, Figure 5, Figure 6 and Figure 7.

### 4.3. Evaluation Metrics

In our previous studies [52,53,54,55,56,57,58], we computed metrics such as the F-measure (FM), precision, and recall. The FM is the weighted average that balances the measurements between the precision and recall rates. The precision is the ratio of the number of correctly predicted positive observations to total number of predicted positive observations. The recall is the ratio of the number of correctly predicted positive observations to total number of observations in the actual class, as indicated in Equation (4). The following equations can be used to calculate the average precision and recall rates of face recognition methods:

(4) $$Precision=\frac{TP}{TP+FP}\;Recall=\frac{TP}{TP+FN}$$

where *TP* denotes the number of true positives, *FP* denotes the number of false positives, and *FN* denotes the number of false negatives.

The *FM* is calculated using Equation (5), which considers both the precision and recall:

(5) $$FM=\frac{2\times precision\times recall}{precision+recall}$$

The average *FM*, *recall*, and precision of the proposed method were 95.6%. False detection occurred in 4.4% of cases by poor lighting or low-quality images. Wearing of facial masks has been indispensable during the COVID-19 pandemic, and, at the same time, it made the process of facial recognition more difficult [59]. The range of the model accuracy was between 0 and 1, and the metric estimation scores reached their best values at 1. An evaluation of our method and other recently published face detection and recognition methods is presented in Table 4.

**Figure 8:**
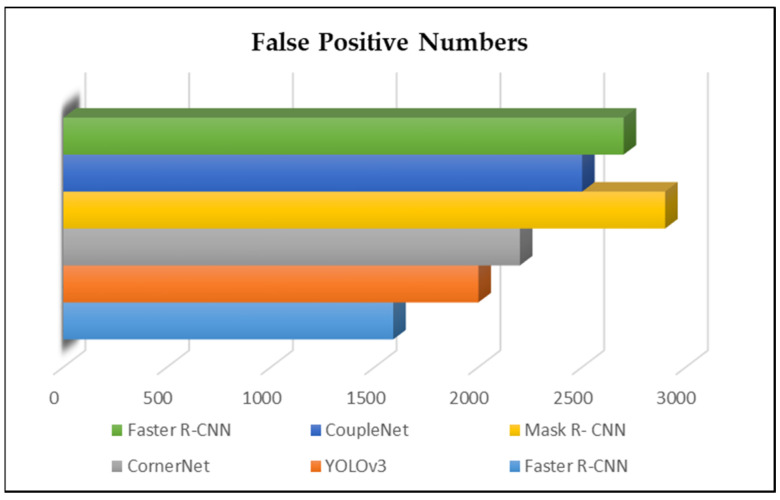
Visible results of false positive speech signal feature extraction experiments.

Furthermore, we assessed the false positive findings of the selected approaches. As seen in Figure 8, the proposed method had the fewest mistakes (error rate). Additionally, the highly efficient multi-scale inference strategy detectors significantly reduced face detection and classification errors. Overfitting was a major concern during training, and it affects nearly all deep learning models. We tried to reduce overfitting risk using data augmentation methods to increase the training data and applying feature selection techniques by choosing the best features and removing the useless/unnecessary features [60,61,62,63,64].

## 5. Conclusions

In this work, we introduced a novel deep learning-based face detection technique. In addition, we used it essentially includes two components: first, a region-offering network (RON) for producing a list of area proposals that, in all likelihood, include faces or regions of interest (RoIs); and second, a prediction network for classifying an area of the image into faces and refining the bounds of these areas. These components contribute common parameters to the feature extraction convolution layers, enabling this architecture to perform face detection tasks at a competitive rate. We used the WIDER FACE dataset to train our model, while the results show that our method is a strong choice for face identification because it can achieve higher accuracy with minimal model size and effective computation. In experiments, we used two popular datasets, such as WIDER FACE and FDDB. Specifically, on the WIDER FACE benchmark, our proposed method achieves AP of 41.0 at speed of 11.8 FPS with a single-scale inference strategy and AP of 44.2 with a multi-scale inference strategy, which are results among one-stage detectors. Then, we trained our model during the implementation using the PyTorch framework, which provided an accuracy of 95% for the faces, which are successfully detected. The results show that our method is a strong choice for face identification because it can achieve higher accuracy with minimal model size and effective computation.

Future tasks include solving blurry image problems under dark conditions and increasing the accuracy of the approach. We plan to develop a small real-time model with a reliable landmark-based face emotion recognition performance employing a variety of datasets in 3D CNN, 3D U-Net, and YOLOv environments.

## Figures and Tables

**Table 1:** Assigning difficulty values: a strategy.

Blur	Expression	Illumination		Occlusion		Pose
Normal Blur	Heavy Blur	Severe Expression	Severe Illumination	limited Occlusion	Hard Occlusion	Unusual Pose
0.5	1	1	1	0.5	1	1

**Table 2:** Speed-accuracy curve compared with other techniques.

Method	mAP	Time
[A] YOLO3–608	33.0	51
[B] SSD–321	28.0	61
[B *] SSD–321	28.2	22
[C] DSSA-321	28.0	85
[D] R-FCN	29.9	85
[E] SSD–513	31.2	125
[E*] SSD–513	31.0	37
[F] DSSD–513	33.2	156
[G] FPN FRCN	36.2	172
[H *] CornerNet	40.5	228
RetinaNet	39.1	198
[*] RefineDet	36.7	110
Ours	41.0	84.7

[*] Tested on our machine for fair comparison.

**Table 3:** State-of-the-art methods comparisons.

Method	Backbone	Input Size	MultiScale	FPS	Avg. Precision, IoU:			Avg. Precision, Area:		
					0.5:0.95	0.5	0.75	S	M	L
two-stage:										
Faster R-CNN (Ren et al., 2015)	VGG-16	~1000 × 600	False	7	21.9	42.7	-	-	-	-
OHEM ++ (Shrivastava et al., 2016)	VGG-16	~1000 × 600	FALSE	7	25.5	45.9	26.1	7.4	27.7	40.3
R-FCN (Dai et al., 2016)	ResNet-101	~1000 × 600	FALSE	9	29.9	51.9	-	10.8	32.8	45.0
CoupleNet (Zhu et al., 2017)	ResNet-101	~1000 × 600	FALSE	8.2	34.4	54.8	37.2	13.4	38.1	50.8
Faster R-CNN w FPN (Lin et al., 2017a)	Res101-FPN	~1000 × 600	FALSE	6	36.2	59.1	39.0	18.2	39.0	48.2
Deformable R–FCN (Dai et al., 2017)	Inc-Res-v2	~1000 × 600	FALSE	-	37.5	58.0	40.8	19.4	40.1	52.5
Mask R-CNN (He et al., 2017)	ResNeXt-101	~1280 × 800	FALSE	3.3	39.8	62.3	43.4	22.1	43.2	51.2
Fitness–NMS (Tychen–Smith and Petersson 2018)	ResNet-101	~1024 × 1024	True	5	41.8	60.9	44.9	21.5	45.0	57.5
Cascade R-CNN (Cai and Vasconcelos 2018)	Res101-FPN	~1280 × 800	FALSE	7.1	42.8	62.1	46.3	23.7	45.5	55.2
SNIP (Singh and Davis 2018)	DPN-98	-	TRUE	-	45.7	67.3	51.1	29.3	48.8	57.1
one–stage:										
SSD300*(Liu et al., 2016)	VGG-16	300 × 300	FALSE	43	25.1	43.1	25.8	6.6	25.9	41.4
RON384++ (Kong et al., 2017)	VGG-16	384 × 384	FALSE	15	27.4	49.5	27.1	-	-	-
DSSD321 (Fu et al., 2017)	ResNet-101	321 × 321	FALSE	9.5	28.0	46.1	29.2	7.4	28.1	47.6
RetinaNet 400(Lin et al., 2017b)	ResNet-101	~640 × 400	FALSE	12.3	31.9	49.5	34.1	11.6	35.8	48.5
RefineDet320(Zhang et al., 2018)	VGG-16	320 × 320	FALSE	38.7	29.4	49.2	31.3	10.0	32.0	44.4
RefineDet320(Zhang et al., 2018	ResNet-101	320 × 320	TRUE	-	38.6	59.9	41.7	21.1	41.5	47.6
Ours	VGG-16	320 × 320	FALSE	33.4	33.5	52.4	35.6	14.4	37.6	47.6
Ours	VGG-16	320 × 320	TRUE	-	38.9	59.1	42.4	24.4	41.5	47.6
Ours	ResNet-101	320 × 320	FALSE	21.7	34.3	53.5	36.5	14.8	38.8	47.9
Ours	ResNet-101	320 × 320	TRUE	-	39.7	60.0	43.3	25.3	42.5	48.3
YOLOV3 (Redmon and Farhadi 2018)	DarkNet-53	608 × 608	FALSE	19.8	33.0	57.9	34.4	18.3	35.4	41.9
SSD512* (Liu et al., 2016)	VGG-16	512 × 512	FALSE	22	28.8	48.5	30.3	10.9	31.8	43.5
DSSD513 (Fu et al., 2017)	ResNet-101	513 × 513	FALSE	5.5	33.2	53.3	35.2	13.0	35.4	51.1
RetinaNet500 (Lin et al., 2017b)	ResNet-101	~832 × 500	FALSE	11.1	34.4	53.1	36.8	14.7	38.5	49.1
RefineDet512 (Zhang et al., 2018)	VGG-16	512 × 512	FALSE	22.3	33.0	54.5	35.5	16.3	36.3	44.3
RefineDet512 (Zhang et al., 2018)	ResNet-101	512 × 512	TRUE	-	41.8	62.9	45.7	25.6	45.1	54.1
CornerNet (Law and Deng 2018)	Hourglass	512 × 512	FALSE	4.4	40.5	57.8	45.3	20.8	44.8	56.7
CornerNet (Law and Deng 2018)	Hourglass	512 × 512	TRUE	-	42.1	57.8	45.3	20.8	44.8	56.7
Ours	VGG-16	512 × 512	FALSE	18	37.6	56.6	40.5	18.4	43.4	51.2
Ours	VGG-16	512 × 512	TRUE	-	42.9	62.5	47.7	28.0	47.4	52.8
Ours	ResNet-101	512 × 512	FALSE	15.8	38.8	59.4	41.7	20.5	43.9	53.4
Ours	ResNet-101	512 × 512	TRUE	-	43.9	64.4	48.0	29.6	49.6	54.3
RetinaNet800 (Lin et al., 2017b)	Res101–FPN	~1280 × 800	FALSE	5	39.1	59.1	42.3	21.8	42.7	50.2
Ours	VGG-16	800 × 800	FALSE	11.8	41.0	59.7	45.0	22.1	46.5	53.8
Ours	VGG-16	800 × 800	True	-	44.2	64.6	49.3	29.2	47.9	55.1

**Table 4:** Quantitative accuracy results of face detection and recognition methods.

Algorithm	Precision	Recall	FM	Average
Faster R-CNN	0.834	0.939	0.902	0.891
CoupleNet	0.968	0.881	0.921	0.923
Mask R-CNN	0.801	0.877	0.883	0.853
CornerNet	0.911	0.904	0.912	0.909
YOLOv3	0.941	0.929	0.932	0.934
Proposed method	0.954	0.958	0.956	0.956

## Data Availability

Data sharing not applicable.
